# Oncological Outcome for 83 Consecutive Patients With Malignant Peripheral Nerve Sheath Tumors Treated at a Tertiary Referral Centre

**DOI:** 10.1002/cnr2.70406

**Published:** 2025-11-21

**Authors:** Hannah V. M. Yeomans, Kira C. Lloyd, Felix Haglund de Flon, Sharmineh Mansoori, Panagiotis Tsagkozis, Christina M. Linder Stragliotto

**Affiliations:** ^1^ Department of Breast Cancer, Endocrine Tumours and Sarcoma Karolinska University Hospital Stockholm Sweden; ^2^ Department of Pathology and Cancer Diagnostics Karolinska University Hospital Stockholm Sweden; ^3^ Department of Oncology‐Pathology Karolinska Institutet Stockholm Sweden; ^4^ Department of Medical Sciences Uppsala University Uppsala Sweden; ^5^ Department of Molecular Medicine and Surgery Karolinska Institutet Stockholm Sweden; ^6^ Department of Acute and Reparative Medicine Karolinska University Hospital Stockholm Sweden

**Keywords:** MPNST, neurofibromatosis type 1, oncological treatment, soft tissue sarcoma

## Abstract

**Background:**

Malignant peripheral nerve sheath tumors (MPNSTs) are rare soft tissue sarcomas with a high risk of recurrence and a poor prognosis, and there is a lack of knowledge regarding long‐term follow‐up and response to oncological treatment.

**Aims:**

The aim of this study was to investigate what treatment the patients received and to examine the outcome for patients with MPNST.

**Methods and Results:**

This is a retrospective study of patients treated for MPNST at Karolinska University Hospital between 2003 and 2022. Data regarding surgical and oncological treatment and follow‐up were collected. Eighty‐three patients were identified and included in the study. Tumor grade is available for 72 patients, of which 64 had high‐grade tumors. Seventy‐nine patients were primarily operated on. Twelve patients presented with distant metastases at diagnosis. Another 37 patients developed local recurrence or distant metastases during follow‐up; the median time from surgery to recurrence was 10.5 months (1–95 months, *n* = 36). The overall mortality rate during the study period was 44% (*n* = 82). Twenty‐seven patients received palliative systemic treatment. The most used therapy for first‐line palliative systemic treatment was doxorubicin and ifosfamide. The disease control rate for first‐line treatment was 33% (*n* = 21). The mean overall survival for the cohort was 132 months (95% CI 107–157 months).

**Conclusion:**

Forty‐five percent of the patients in this material were diagnosed with recurrent disease and most patients treated with palliative systemic therapy experienced brief disease control following treatment. Among patients with MPNST treated with first‐line palliative oncological treatment, doxorubicin and ifosfamide have the highest disease control rates. The study also identified a few patients with long‐term treatment responses, with four patients alive more than 2 years after starting palliative oncological treatment.

**Abbreviations**
CR: complete response
CRR: clinical response rate
DCR: disease control rate
DFS: disease‐free survival
DMFS: distant metastasis free survival
FNCLCC: Fédération Nationale des Centres de Lutte Contre le Cancer
LRFS: local recurrence free survival
MPNST: malignant peripheral nerve sheath tumor
NF1: neurofibromatosis 1
OS: overall survival
PD: progressive disease
PFS: progression free survival
PR: partial response
RECIST: response evaluation criteria in solid tumors
RR: response rate
SBRT: stereotactic body radiation therapy
SD: stable disease
SSG: Scandinavian Sarcoma Group

## 1 Introduction

Malignant peripheral nerve sheath tumor (MPNST) is a type of soft‐tissue sarcoma [1, 2]. Most MPNSTs are thought to be of neural crest origin [1, 3]. Approximately 50% of cases arise in patients with neurofibromatosis type 1 (NF1), an autosomal dominant genetic disorder in which mutations are found in the Neurofibromin 1 gene [1, 4]. In patients with MPNST, inactivating mutations in the Neurofibromin 1 gene may cause loss of neurofibromin (Ras‐inhibiting enzyme); activation of Ras and downstream pathways causes upregulation of PI3K/AKT/mTOR/MAPK signal transduction, which for example regulates tumor proliferation and angiogenesis [5]. The birth incidence of NF1 is approximately 1:3000 [6]. The lifetime risk of developing MPNST is 8%–13% among NF1 patients [1, 5, 7], and the diagnosis of MPNST contributes considerably to a reduced life span in these patients [8]. It is also possible that NF1 is underdiagnosed [9]. Another risk factor for developing MPNST is radiation exposure [1, 4, 10].

The tumor typically debuts as a growing mass, commonly situated near nerve roots and bundles of the extremities and the pelvis [1]. Pain, paraesthesia, and neurological deficits are common symptoms [1, 11]. Surgical resection with wide negative margins and adjuvant radiotherapy is the clinical standard of care for localized high‐grade MPNST [1, 4]. Forty to 65% of patients suffer local recurrence, and 30%–60% develop metastases (most commonly in the lungs) [1]. More than 10% of MPNST patients have non‐resectable tumors or metastatic disease at diagnosis [4]. The first‐line palliative treatment consists of a chemotherapy regimen containing anthracycline (doxorubicin or epirubicin). Combined doxorubicin and ifosfamide, alternatively doxorubicin monotherapy, is commonly used as first‐line treatment [4]. Treatment with the combination of doxorubicin and ifosfamide has shown a clinical response rate (CRR) of approximately 21% [4, 12]. The addition of ifosfamide has been shown to improve the progression‐free survival (PFS) but not the overall survival (OS), although it often causes higher toxicity [4, 13].

MPNST is an aggressive disease with a poor prognosis [1, 10, 11, 14, 15]. The overall 5‐year survival ranges from 20% to 50% in patients with high‐grade MPNST [1]. Since MPNST is rare [10], there is a need for further knowledge.

The aim of this study was to examine the outcome for patients with MPNST treated at Karolinska University Hospital between 2003 and 2022, and to investigate how patients responded to given surgical and oncological treatment. The main hypothesis was that the outcome and response to oncological treatment are poor in patients with MPNST.

## 2 Methods

### 2.1 Study Design

The study is a retrospective cohort study of patients treated for MPNST at the Sarcoma center of the Karolinska University Hospital, a tertiary referral hospital in Stockholm, Sweden. Due to its retrospective design and long timeframe, no written informed consent was collected from patients. The study has been approved by the Swedish Ethical Review Authority (reference number 2022‐05409‐01).

### 2.2 Setting and Participants

Cases of MPNST were identified through the digital archive of the Pathology department, including cases between January 2003 and May 2023 (*n* = 93). All pathology reports were reviewed and patients with uncertain diagnoses were excluded. The minimum follow‐up period after surgery was set at 6 months, and alive patients with a shorter follow‐up were excluded. Patients with recurrent MPNST originally diagnosed before 2003 were also excluded. Data regarding tumor characteristics and genetics were collected from the pathology reports; information regarding surgical and oncological treatment as well as follow‐up was compiled from the patients' electronic medical records, see Table S1. Where applicable, treatment details were obtained from other hospitals.

In the present material, tumor grades were reported according to the Fédération Nationale des Centres de Lutte Contre le Cancer (FNCLCC) histological grading system (I–III) and/or the older Scandinavian Sarcoma Group (SSG) grading system (I–IV). Tumors with grade II and III according to FNCLCC and grade III and IV according to SSG were classified as “high grade.” Tumors over 8 cm were considered large, in line with the SSG study SSG XX‐protocol [16]. In cases where tumor size was not stated in the pathology report, information from radiology reports or ultimately palpatory measurements was used.

OS was calculated from the date of diagnosis, defined as the date of the pathology report, to the date of death or the date of cut‐off for data collection (set as July 5th, 2023). Disease‐free survival (DFS) was calculated from the time of diagnosis to the time of local recurrence, metastasis (whichever came first), or death. For patients receiving palliative treatment, PFS was defined as the time from the start of treatment to radiological signs of progression. Radiological evaluation was not performed according to RECIST (Response Evaluation Criteria in Solid Tumors) or at fixed time points due to the retrospective design of the study. Instead, radiological examinations were evaluated according to local routines for patients not participating in clinical trials at the Karolinska University Hospital. Complete response (CR) indicated the absence of any visible tumor, partial response (PR) denoted a visible reduction in size compared to the lesion before treatment, and stable disease (SD) indicated no apparent change in size. Disease control rate (DCR) was used to evaluate response to treatment and included patients with CR, PR, and SD. Progressive disease (PD) was defined as a visible increase in the cross‐sectional tumor diameter.

### 2.3 Statistical Analysis

Survival analysis was performed as per Kaplan and Meier, and the log rank test was used for comparison between groups. Correlation between patient survival time and possible prognostic factors was done in a multivariable Cox regression model with hazard ratios (HRs) and 95% confidence intervals (CIs). *p*‐values < 0.05 were considered statistically significant. Statistical analysis was performed in SPSS 25 (IBM, NY).

## 3 Results

### 3.1 Description of the Cohort

**FIGURE 1:**
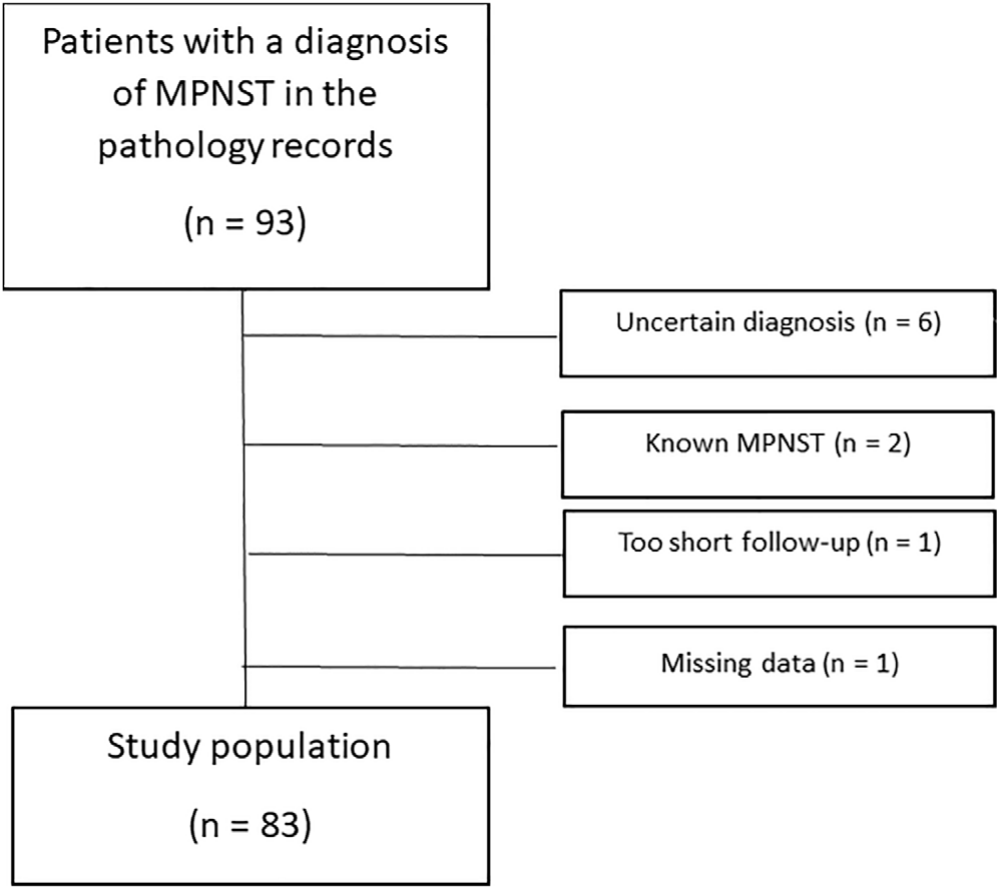
Patient flow chart. Inclusion of patients to the study population.

Patients with a pathology report with the diagnosis of MPNST between January 2003 and May 2023 (*n* = 93) were considered eligible for inclusion. Patients with an uncertain diagnosis (*n* = 6), diagnosis before 2003 (*n* = 2), missing data (*n* = 1) or follow‐up shorter than 6 months (*n* = 1) were excluded, resulting in a study population of 83 patients (see Figure 1).

The patient and tumor characteristics of the study population are listed in Table 1, including a comparison between patients with and without NF1.

**TABLE 1:** Patient and tumor characteristics and outcome of the cohort.

Variable	Total study population	Patients with NF1	Patients without NF1
	*n* = 83	*n* = 19	*n* = 64
Mean age	53 (range 9–89, median 54) years	37 (range 9–66, median 36) years	57 (range 10–89, median 60) years
Under age 40	22 (27%)	11 (58%)	11 (17%)
Women	42 (51%)	8 (42%)	34 (53%)
Primary tumor location			
Extremity	50 (60%)	12 (63%)	38 (59%)
Trunk	30 (36%)	7 (37%)	23 (36%)
Head/neck	3 (4%)	0 (0%)	3 (5%)
NF1^a^	19 (23%)		
Possible radiation‐induced tumor^b^	6 (7%)	1 (5%)	5 (8%)
Multiple malignancies^c^	20 (24%)	5 (26%)	15 (24%)
Surgery	79 (95%)	19 (100%)	60 (94%)
High tumor grade	64 (89%) (*n* = 72)^d^	17 (94%) (*n* = 18)^d^	47 (87%) (*n* = 54)^d^
Tumor size > 8 cm	32 (46%) (*n* = 70)^d^	10 (56%) (*n* = 18)^d^	22 (42%, *n* = 52)
Surgical margin			
Wide	21 (28%, *n* = 75)	6 (32%, *n* = 19)	15 (27%, *n* = 56)
Marginal	32 (43%, *n* = 75)	9 (47%, *n* = 19)	23 (41%, *n* = 56)
Intralesional	22 (29%, *n* = 75)	4 (21%, *n* = 19)	18 (32%, *n* = 56)
N/A^e^	8 (11%)	—	8 (13%, *n* = 64)
Extended surgical excision	15 (19%) (*n* = 80)^d^	5 (26%)	10 (16%) (*n* = 61)^d^
Radiotherapy (neoadjuvant or adjuvant)	40 (49%) (*n* = 82)^d^	11 (58%)	29 (46%) (*n* = 63)^d^
Neoadjuvant chemotherapy	3 (4%)	1 (5%)	2 (3%)
Adjuvant chemotherapy	5 (6%)	1 (5%)	4 (6%)
DM (distant metastases) at baseline	12 (14%)	3 (16%)	9 (14%)
Recurrent disease	37 (45%)	8 (42%)	29 (45%)
Local recurrence	28 (34%)	8 (42%)	20 (31%)
DM (baseline and follow‐up)	36 (43%)	10 (53%)	26 (41%)
DM at follow‐up	24 (29%)	7 (37%)	17 (27%)
Suspected DM, ongoing investigation	1 (1%)	1 (5%)	0
Lost to follow‐up	6 (7%)	1 (5%)	5 (8%)
Relapse‐free	34 (41%)	8 (42%)	26 (41%)

*Note:* Data collected at the time of primary diagnosis of MPNST and during follow‐up for the total study population (*n* = 83), patients with NF1 (*n* = 19) and patients without NF1 (*n* = 64).

Abbreviation: DM, distant metastases.

^a^ Not all patients were tested for NF1.

^b^ These patients had all received radiation therapy in the same area due to another malignancy.

^c^ Diagnosed before or after MPNST.

^d^ In these cases, data was not available in the medical journals of all patients.

^e^ Data missing (*n* = 5) or patient not operated.

### 3.2 Patient Survival and Associated Factors

**FIGURE 2:**
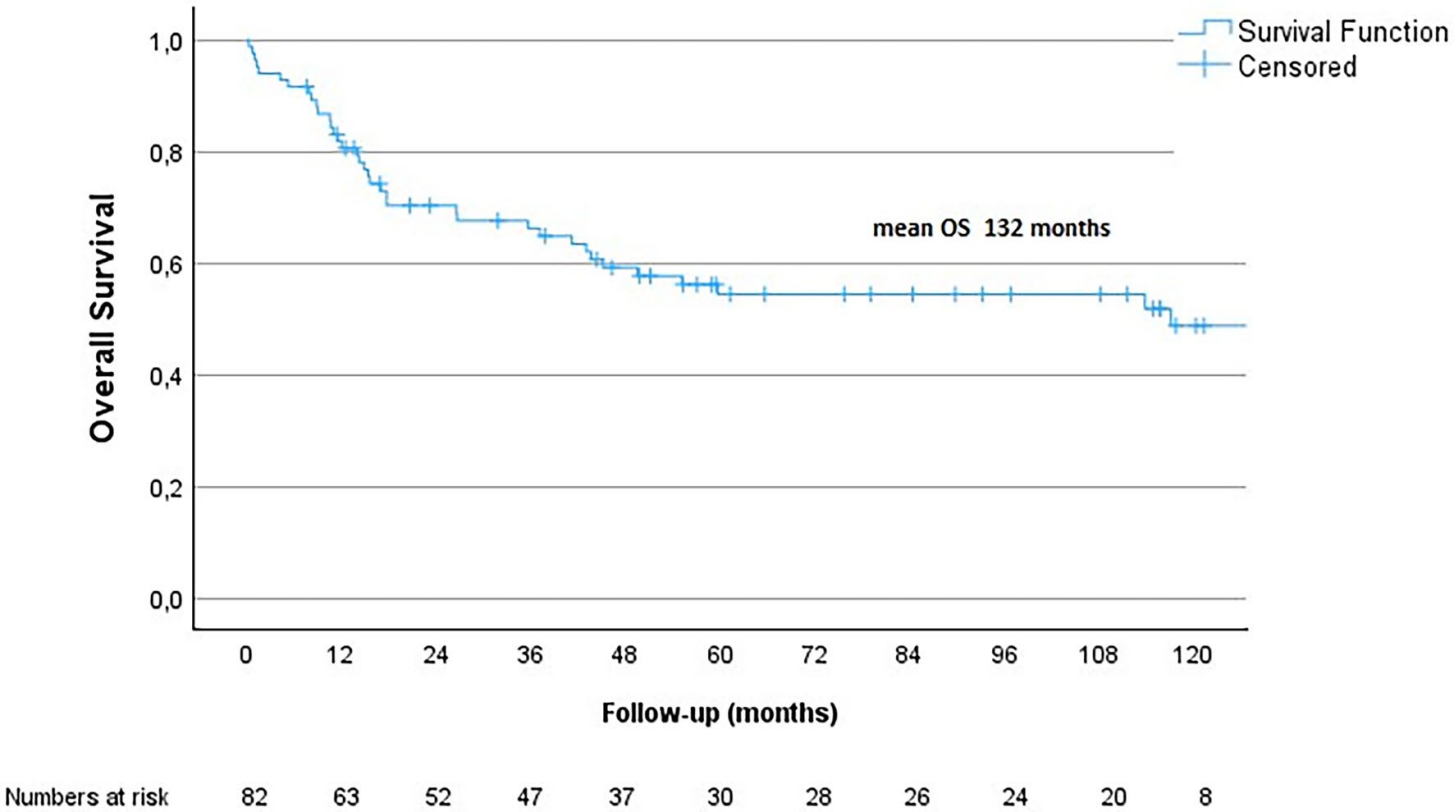
Overall Survival. OS for the study population.

Five‐year OS was 54% and mean OS was 132 months (95% CI 107–157 months), see Figure 2. Mean OS for patients without metastases at diagnosis was 148 months (95.0% CI 121–174 months) and for those initially diagnosed with metastatic disease 22 months (95.0% CI 6–39 months). The overall mortality rate during the study period was 44% (*n* = 82). Cox regression was performed for tumor size and tumor grade, with further adjustments for age and sex, as well as separately for NF1 status and the presence of metastases at diagnosis. As described in Table 2, large tumor size was associated with worse OS. Furthermore, as expected, patients with metastatic disease at diagnosis had a worse outcome. There was no significant correlation between tumor grade, age, NF1 status or gender and OS.

**TABLE 2:** Hazard ratios for the event of death.

	*p*	HR	95% CI for HR	
			Lower	Upper
Size (cm)	**0.000**	1.143	1.071	1.221
Grade (high)	0.442	2.242	0.287	17.541
Age (years)	0.311	1.014	0.987	1.040
Sex (man)	0.928	1.041	0.440	2.460
NF1 status (presence)	0.443	1.618	0.474	5.527
Metastasis at diagnosis (presence)	**0.009**	4.689	1.463	15.024

*Note:* COX regression analysis for tumor size and tumor grade with adjustments for age and sex. Bold font indicates a significant *p*‐value. Analysis was done separately including metastasis at diagnosis as a covariable.

Abbreviations: CI, confidence interval; cm, centimeters; HR, hazard ratio.

**FIGURE 3:**
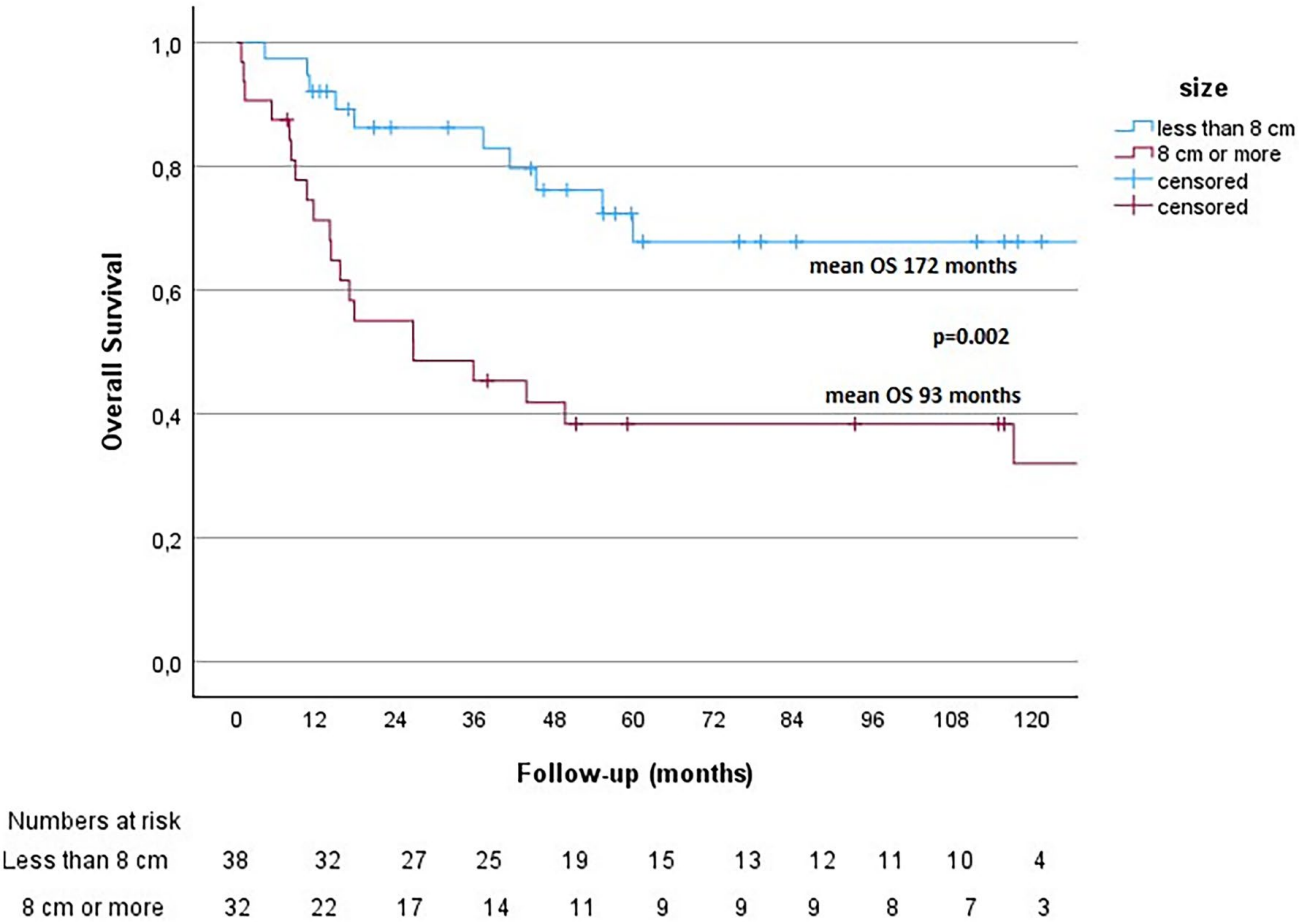
Size and OS. Kaplan–Meier survival analysis of the association between size and OS.

Tumor size and its association with OS was further subject to Kaplan–Meier survival analyses, as shown in Figure 3. Log‐minus‐log analysis showed that tumor size analysis met the criteria for the proportional hazard assumption.

### 3.3 Local and Distant Relapse of the Disease

**FIGURE 4:**
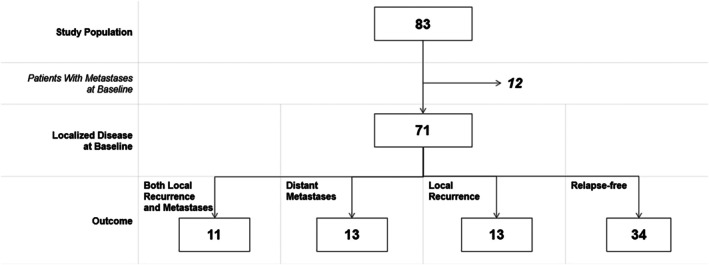
Outcome. Patients with localized and metastatic disease at baseline and during follow‐up.

From the study population of 83 patients, 12 patients had distant metastases at baseline. Of the remaining 71 patients with localized disease, 34 remained relapse‐free during follow‐up and 37 relapsed, either locally and/or with distant metastases (see Figure 4). The median time from surgery to recurrence was 10.5 months (1–95 months, *n* = 36).

Among patients with metastatic disease, the primary site of metastasis was known for 34 patients: lung in 21 patients (61%), soft tissue in three patients (9%), bone in three patients (9%), multiple sites in two patients (6%), lymph nodes in two patients (6%), extremity in one patient (3%), pericardium in one patient (3%), and abdomen (carcinomatosis) in one patient (3%). Five patients could not be followed for the duration of the palliative period but are known to have had metastatic or recurrent disease and are therefore included in the categories above. The median follow‐up time for the cohort was 46 months (range: 0–239 months). The outcome of the follow‐up of patients with MPNST is presented in Table 1. The DFS for patients without metastases at diagnosis was 96 months (95% CI 70–122 months). For DFS for the entire cohort, please refer to Figure S1.

**FIGURE 5:**
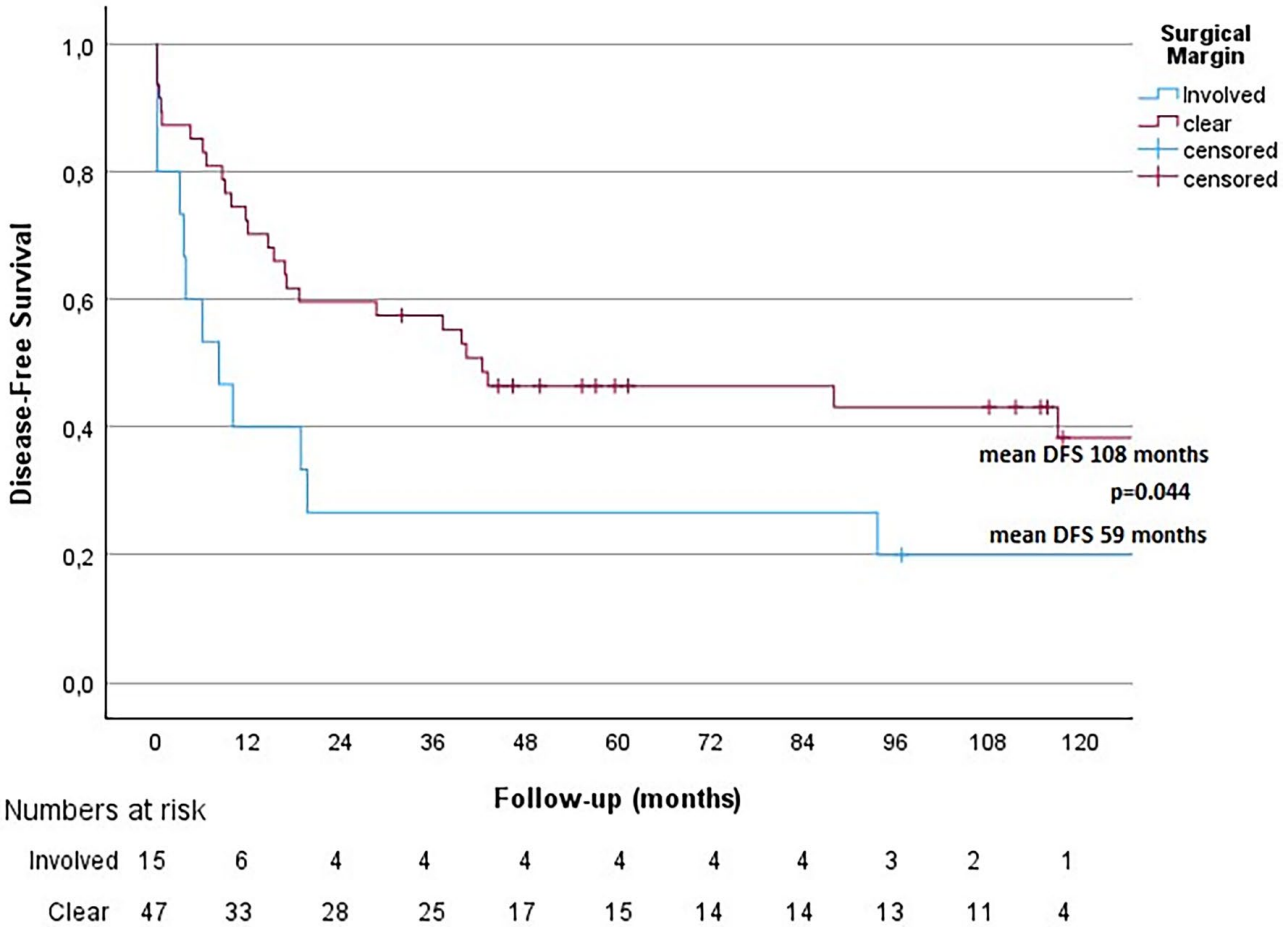
Disease‐free survival and surgical margin. Kaplan–Meier survival analysis of the association between surgical margin and DFS. An *involved* margin constitutes tumors operated with an intralesional margin (R1). A clear margin constitutes tumors operated with a marginal or *wide* margin (R0).

Clear surgical margin (wide or marginal) was associated with superior disease‐free survival (*p* = 0.044), see Figure 5, but not significantly superior OS (*p* = 0.139). Of 28 patients with diagnosed local recurrence (this number including patients with metastases at diagnosis), data were available for 27 patients, of which 22 underwent surgical resection. Of these 22 patients, six have ongoing follow‐up without signs of relapse (median follow‐up 72 months, range 22–107 months). Fourteen patients relapsed after surgical resection: with new local recurrence (*n* = 4), distant metastases (*n* = 8), or both (*n* = 2). One additional patient was operated on suspicion of a new local recurrence where pathological diagnosis was inconclusive; the tumor was malignant but may be a recurrence of MPNST or a radiation‐induced sarcoma of another type. One patient had ongoing post‐operative radiotherapy at cut‐off for data collection.

Regarding the 36 patients with diagnosed distant metastases at baseline or during follow‐up, data is available for 33 patients of which 15 underwent surgical resection of lung metastasis (*n* = 8), bone metastasis (*n* = 2), both lung and bone metastasis (*n* = 2), metastasis to the pericardium (*n* = 1), soft tissue metastasis (*n* = 1) or not specified (*n* = 1). Of these 15 patients, 12 later died of progressive disease. At cut‐off for data collection two patients were relapse‐free under ongoing follow‐up (median follow‐up: 36 months, range: 24–48 months, *n* = 2). One additional patient was alive but under investigation due to suspected recurrent disease at cut‐off for data collection.

### 3.4 Palliative Oncological Treatment

**FIGURE 6:**
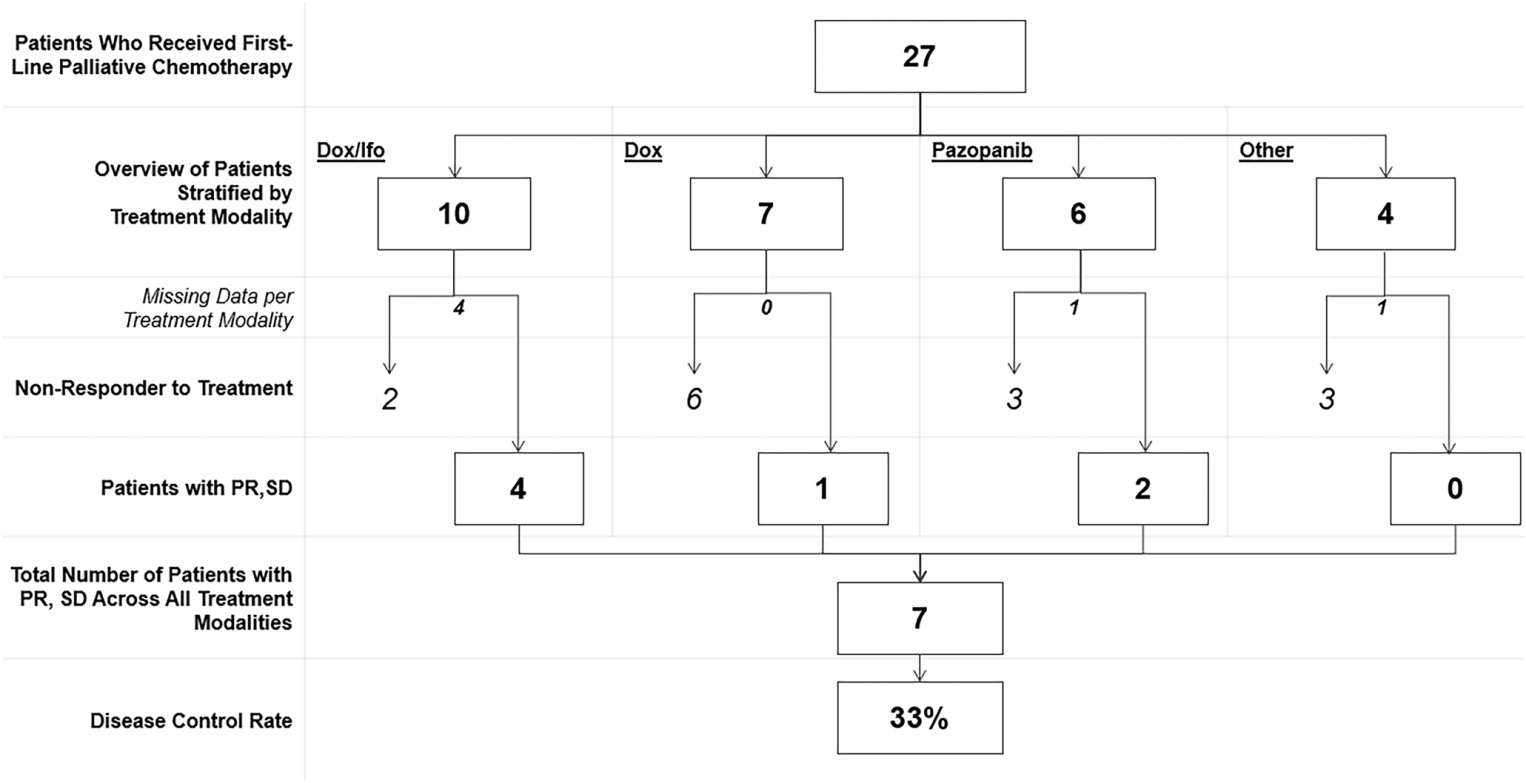
Patients who received first‐line palliative chemotherapy stratified by treatment modality and response with PR or SD. These patients included patients diagnosed with metastases at baseline and during follow‐up. Dox, doxorubicin; Ifo, ifosfamid; PR, partial response; SD, stable disease.

Twenty‐seven patients in this study are known to have received palliative oncological treatment for MPNST, see Table 3. For the 27 patients given first‐line treatment, evaluation of response was possible for 21 patients, of which seven had stable disease or regression at first radiological evaluation (disease control rate [DCR]: 33%), see Figure 6.

**TABLE 3:** Systemic treatment.

Systemic treatment	1st line	2nd line	3rd line	4th line	5th line	6th line
Doxorubicin + ifosfamide^a^	10					
Doxorubicin	7					
Pazopanib	6	5	3		1	
Liposomal doxorubicin^b^	2			1	1	
Temozolomide	1					
Doxorubicin + ifosfamide + vincristine	1					
Docetaxel + gemcitabine		1	2			
Trabectedin		2				
Trametinib + abemaciclib				1		
Eribulin		1		1		
Pegylated liposomal doxorubicin + ifosfamide		1				
Trofosfamide						1
Carboplatin + etoposide		1				
Pembrolizumab			1			

*Note:* Number of patients with palliative systemic treatment in the first to sixth line.

^a^ For two patients, ifosfamide was added to the second (*n* = 1) or third (*n* = 1) treatment. One patient received an additional two treatments with pegylated liposomal doxorubicin and ifosfamide after reaching maximum dose of doxorubicin.

^b^ Unpegylated liposomal doxorubicin (*n* = 1), pegylated liposomal doxorubicin (*n* = 1).

Among the 10 patients treated in the first line with doxorubicin and ifosfamide, six patients were available for evaluation of response. Four out of six patients responded with PR or SD. Estimation of PFS is possible for eight patients (mean PFS: 15.5 weeks [95.0% CI 6.6–24.4 weeks], *n* = 8). In two out of four patients who responded with PR or SD to treatment with doxorubicin and ifosfamide, treatment was interrupted due to maximum cumulative dose of doxorubicin, which complicates analysis of PFS.

Out of seven patients treated with single doxorubicin in the first line, one patient responded to treatment with PR or SD. For patients treated with pazopanib in the first line, five out of six patients are available for evaluation of response; two out of five patients responded to treatment with PR or SD.

For the following lines of palliative systemic treatment, fewer patients were available for evaluation. From the 27 patients treated in the first line, nine were deceased in connection to treatment. Three patients did not receive second‐line treatment for unknown reasons but were deceased within 1 year after ending first line. One patient was stable without treatment at cut‐off for data collection. One patient was treated with SBRT and surgical excision. One patient was lost to follow‐up. One patient had just begun treatment at cut‐off for data collection.

#### 3.4.1 Long Term Survivors

For patients given first‐line palliative systemic treatment, survival from the commencement of treatment is known for 26 patients; nine of these patients (35%) lived more than 1 year after starting palliative treatment. Four patients (15%) lived more than 2 years after starting palliative systemic treatment.

One patient who is a long‐term survivor (treatment nearly 7 years after diagnosis of distant metastases) has ongoing sixth‐line palliative treatment for MPNST. Another patient who did not respond to treatment with doxorubicin in the first line was treated with surgical excision and SBRT and is still relapse‐free after 5 years of follow‐up. An additional patient who had received several lines of palliative treatment prior was treated with CDK 4/6 inhibitor abemaciclib and MEK inhibitor trametinib in the fourth line after molecular testing and a review of the literature and current case reports were performed. Ten weeks into treatment, evaluating radiology showed a combination of response and mixed response. The patient developed side effects with skin rash causing temporary interruption of trametinib, which was later restarted in a lower dose. Follow‐up radiology at 21 weeks of treatment showed progression and the treatment was interrupted.

## 4 Discussion

This study of 83 patients with MPNST with a long follow‐up time showed a median OS of 117 months. Previous studies have shown median OS periods of 44–132.5 months [9, 15, 17]. Tumor size and quality of margins [15, 18] as well as NF1 [18] have in some previous studies been shown to significantly affect OS. In the present material, tumor size over 8 cm was a significant prognostic factor negatively affecting OS. Other recent studies have shown a correlation between high‐grade tumors (according to FNCLCC) and shorter survival [10, 19, 20]. The present study could not show a significant correlation between tumor grade and OS, probably because there were few cases with low‐grade tumors in the material. The impact of negative surgical resection margins is well established regarding local control of the disease in soft tissue sarcomas but still remains a matter of debate regarding overall patient survival [21, 22]. In this study, we could not see an association between surgical margin and OS. The size of the cohort may also play a role in this.

From the study population of 83 patients, only 19 (23%) had confirmed NF1, compared to other studies showing an incidence of NF1 of 40%–50% [5, 23]. Most likely, NF1 is underdiagnosed in the present material since NF1 testing was not routinely performed in patients with MPNST until more recently.

During follow‐up, 37 patients in this study (45%) were diagnosed with recurrent disease. However, 8 patients (10%) had follow‐up of less than 2 years at cut‐off for data collection, which implies the number of patients with relapse may increase over time.

Twenty‐seven patients were treated with palliative systemic treatment, where 33% of patients available for evaluation (*n* = 21) responded to first‐line palliative treatment with stable disease or regression. The highest DCR in the first line was seen in patients given the combination of doxorubicin and ifosfamide, although the number of patients available for evaluation was few (*n* = 6). The present material also showed that treatment with pazopanib had a higher DCR than single doxorubicin in the first line. Pazopanib has previously been shown to be noninferior to single doxorubicin in a study of patients with metastatic soft tissue sarcoma age 60 years and older [24]. Another study by Sobczuk et al. [25] showed no significant difference in median PFS between doxorubicin‐based regimens and pazopanib.

The present material showed that very few patients were treated with more than two lines of palliative systemic treatment, although one patient who was a long‐term survivor had ongoing sixth line palliative treatment at cut‐off for data collection. Another patient was treated with CDK4/6 inhibitor abemaciclib and MEK‐inhibitor trametinib in the fourth line. Preclinical studies of MPNST and neurofibroma have suggested investigation of MEK inhibitors in the treatment of Ras‐related diseases, including NF1 [26]. A preclinical study published in 2023 investigated the effects of targeting CDK4/6, MEK and PD‐L1 in MPNST and found that CDK4/6 and MEK inhibitors induced an immune response and anti‐tumor activity, which in turn enhanced the effect of anti‐PD‐L1 immune checkpoint blockade in MPNST [27]. Clinical trials have yet to confirm these findings. Currently, the results of a phase 2 study (SARC031) of MEK inhibitor selumetinib and mTOR inhibitor sirolimus in patients with MPNST are awaited [28, 29].

It has previously been shown that patients with metastatic MPNST may benefit from resection of metastases, especially in the lungs [9, 30, 31]. In the present material, two patients who underwent surgical excision of distant metastasis (both in the lung) were relapse‐free under ongoing follow‐up (median follow‐up: 36 months, range: 24–48 months, *n* = 2). An additional patient who initially received first‐line palliative chemotherapy without response, underwent surgical excision of a local recurrence and SBRT of a pulmonary metastasis and is relapse‐free (follow‐up 62 months).

The present study has several limitations, in part due to its retrospective design and small study population. Many patients were referred to their local hospitals for follow‐up which affected data collection. Radiological evaluation was not performed according to RECIST guidelines due to the retrospective nature of the study, nor at fixed time points. Furthermore, the minimum follow‐up period was set at 6 months, which in turn affects available data for analysis of OS and recurrence of disease.

In conclusion, this retrospective study investigates the outcome for 83 patients diagnosed with and treated for MPNST during a 20‐year period. Forty‐six percent of the patients in this material were diagnosed with recurrent disease and most patients treated with palliative systemic therapy had a brief response with PR or SD to treatment. However, the study also identified a few patients with long‐term treatment response with four patients alive more than 2 years after starting palliative oncological treatment. Three of these four patients presented with metastatic disease and did not undergo primary surgery, why complete pathology reports are lacking thus limiting the possibility of drawing any advanced conclusions. The palliative systemic treatment with the highest DCR in the first line was combination treatment with doxorubicin and ifosfamide.

## Author Contributions

Conceptualization: C.M.L.S., P.T., F.H.F., K.C.L., and H.V.M.Y. Methodology: C.M.L.S., P.T., H.V.M.Y., and K.C.L. Investigation: H.V.M.Y., K.C.L., and S.M. Formal analysis: P.T. Supervision: C.M.L.S. and P.T. Writing – original draft: H.V.M.Y., K.C.L., C.M.L.S., and P.T. Writing – review and editing: all authors.

## Funding

This work was supported by the Bernt Katinas Memorial Fund.

## Ethics Statement

The study has been approved by the Swedish Ethical Review Authority. No written informed consent was collected from patients due to the retrospective design and long timeframe of the study.

## Conflicts of Interest

The authors declare no conflicts of interest.

## Supporting information


**Table S1:** Data collection. Variables collected from the patients' electronic medical records.
**Figure S1:** Disease‐free survival. DFS for the study population.

## Data Availability

The dataset used and analyzed during the current study is available from the corresponding author on reasonable request. The data is not openly accessible for confidentiality reasons due to the rareness of MPNST.
