# Supplementary material for: Oncological Outcome for 83 Consecutive Patients With Malignant Peripheral Nerve Sheath Tumors Treated at a Tertiary Referral Centre
Source: Cancer Rep (Hoboken). 2025 Nov 21;8(11):e70406. doi: 10.1002/cnr2.70406 (PMC12638222; doi:10.1002/cnr2.70406)

1. Supplementary

**Table 1. Data collection.** Variables collected from the patients’ electronic medical records.

| **Patient characteristics** | **Tumour characteristics** | **Therapeutic data** |
| --- | --- | --- |
| Age at diagnosis | Tumour localization (trunk/extremity/head & neck) | Surgical margin |
| Date of diagnosis | Tumour size* | Extended excision |
| Date of first operation | Tumour grade** | Postoperative radiotherapy |
| Gender | Proliferation | Postoperative chemotherapy |
| Known NF1 | Mitotic index | Metastases at diagnosis |
| Heredity | Vascular invasion | Early recurrence (within 18 months) |
| Previous radiotherapy | Necrosis | Local recurrence |
| Other malignancies |  | Metastases |
| Date of death or last known survival |  | Repeated surgery |
|  |  | Palliative chemotherapy (response in weeks) |
|  |  | Palliative radiotherapy |
|  |  | Last follow-up |
|  |  | Overall survival |

*In cases where tumour size was not stated in the pathology report, information from radiology reports or ultimately palpatory measurements were used.
** Tumour grades were reported according to the FNCLCC histological grading system (I-III) and/or the older SSG grading system (I-IV). In the present material, tumours with grade II and III according to FNCLCC and grade III and IV according to SSG were classified as “high grade” (see Section 5.1 and 5.2).

**Figure 1. Disease-Free Survival.** DFS for the study population.


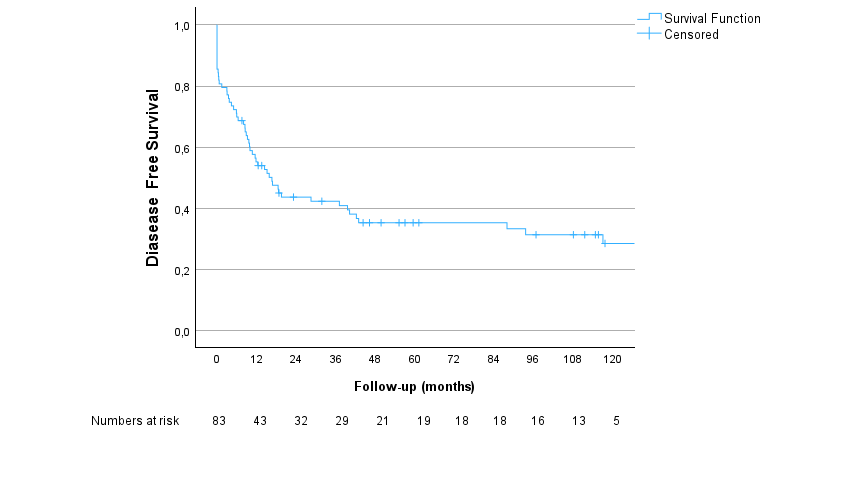

Supplement: Supplementary file 1 — Table S1: Data collection. Variables collected from the patients' electronic medical records. Figure S1: Disease‐free survival. DFS for the study population. [file CNR2-8-e70406-s001.docx]
